# The Condition of the Masseter Muscles After Orthodontic Treatment with Fixed Appliances

**DOI:** 10.3390/diagnostics14232755

**Published:** 2024-12-06

**Authors:** Sebastian Szajkowski, Jarosław Pasek, Grzegorz Cieślar

**Affiliations:** 1 Faculty of Medical and Social Sciences, Warsaw Medical Academy of Applied Sciences, 8 Rydygiera St., 01-793 Warszawa, Poland; sebastianszajkowski@wp.pl; 2 Collegium Medicum im dr Władysława Biegańskiego, Jan Długosz University in Częstochowa, 13/15 Armii Krajowej St., 42-200 Częstochowa, Poland; 3 Department of Internal Medicine, Angiology and Physical Medicine, Faculty of Medical Sciences in Zabrze, Medical University of Silesia in Katowice, 15 Stefana Batorego St., 41-902 Bytom, Poland; cieslar1@o2.pl

**Keywords:** fixed appliances, masseter muscle, bite force, myotonometry, stiffness

## Abstract

Background: One of the methods used in malocclusion treatment is the use of fixed appliances. Research conducted so far has revealed that changes in bite force occurring over the course of orthodontic treatment are directly related to the functional status of the masticatory muscles. It is therefore advisable to find out how the biomechanical parameters of the masseter muscles change after treatment with the application of fixed appliances. Methods: The study material comprised 74 individuals, divided into the study group (*n* = 37) treated by means of fixed orthodontic appliances over the average time of 12.27 months and the control group (*n* = 37) consisting of individuals did not undergo orthodontic treatment. The biomechanical properties of the masseter muscles were examined by means of myotonometry. Results: Upon completion of orthodontic treatment, the values of the parameters of tension, stiffness, and elasticity of masseter muscles located on both sides did not statistically significantly differ between patients from the study group who were treated by means of fixed orthodontic appliances and patients from control group who did not undergo orthodontic treatment. Conclusions: Treatment of malocclusions with the use of fixed appliances does not affect the biomechanical and visco-elastic properties of the masseter muscles (stiffness, tension, and elasticity) assessed by means of myotonometry and it appears safe for masticatory muscles.

## 1. Introduction

Increased awareness of societies regarding the need to take proper care of one’s teeth, in terms of both health and aesthetic aspects, is reflected in the growing popularity of using orthodontic appliances. In orthodontics, treatment planning and treatment progress require the possession of thorough comprehension concerning the functioning of the masticatory muscles and their relationship with facial morphology. One of the malocclusion treatment methods is the application of fixed orthodontic appliances. The force applied to the teeth with their assistance allows the teeth to move in the supporting bone in such directions that result in correction of the occlusion [1]. Orthodontic treatment with the application of fixed appliances begins with leveling of the maxillary and mandibular dental arches using flexible arch wires. This results in the formation of two dental arches and the teeth are then in the correct occlusal contact [2]. Several components influence occlusal bite force; these constituents comprise age, gender, craniofacial morphology, temporomandibular joint disorders, periodontium, and dental status [3]. Bite force appears to be closely related to the number of teeth involved in occlusal contact. Thus, bite force should increase after treatment because of the greater number of teeth that are then in occlusal contact [4]. Changes noted as time elapses, which concern bite force as a result of using fixed appliances, remain inconsistent. It has been noted and described that the bite force used appears to decrease during the initial period of active orthodontic treatment but, as time elapses, its pretreatment levels are restored. It was shown that bite force presented the lowest values one week after installation of the fixed appliance and then increased, reaching pre-treatment levels after 6 months of treatment [5]. These results were not confirmed in a study reported elsewhere, which showed that bite force decreased immediately after the fixed appliance was removed, after 9 months of treatment [6]. Research indicates that the changes occurring in bite force during orthodontic treatment are directly connected with the functional status of muscles of the mastication system. Assessment of bite force and the visco-elastic properties of the masseter muscles are helpful in diagnosing and assessing interference in the stomatognathic system over the course of orthodontic treatment. On the other hand, the short- and long-term effects of this therapy on mastication biomechanics are still unclear [7,8]. Thus, it seems necessary to check the behavior of the biomechanical parameters of the masseter muscles after treatment with the application of fixed orthodontic appliances. Irregularities concerning occlusion occur on average in as many as 56% of members of the general population [9]. Given the high proportion of patients with malocclusion, the need for further knowledge of individual malocclusions and accompanying masseter muscle abnormalities is reasonable. If we want to improve the quality of orthodontic treatment, it is worth referring the patient to a physiotherapist for a functional assessment of the masticatory muscles. Abnormal visco-elastic properties could make it difficult to obtain satisfactory results in orthodontic treatment. Physiotherapy in orthodontics consists of eliminating the symptoms of functional disorders of the masticatory system (excessive tension and stiffness of the masseter muscles) and helps improve the patient’s comfort when using the appliance [10]. Application of botulinum toxin type A (BoNT-A) in the form of injections to the masseter is also practiced for therapeutic and cosmetic purposes as they are virtually non-invasive, while they are highly efficient and their complication rates do not cause concern. (BoNT-A) is known to reduce muscle contraction; consequently, it decreases the forces exerted during episodes of grinding and clenching. (BoNT-A) injection is a procedure that may provide a therapeutic solution for various medical conditions involving the masseter muscles, including malocclusions requiring orthodontic treatment [11].

Both objective and subjective methods are used to assess the performance of the masseter muscles. Objective methods comprise the measurement of maximum bite force [12], efficiency of chewing and swallowing [13], and masseter muscle activity [14]. Subjective methods include the use of self-assessment questionnaires [15]. Bite force assessment is widely used to demonstrate the relationship between masseter muscles and teeth in occlusal contact. On the other hand, the key factor determining the maximum strength that a muscle can generate is its cross-sectional area (CSA). Previous studies have shown that bite force is proportional to the muscle cross-sectional area and is directly linked with the level of stiffness in the myotonometric test carried out in the thickest place of the belly of the masseter muscles [16].

Previous studies provide inconsistent data concerning the effects of the use of orthodontic appliances on masseter muscles, which is thus the basis for exploring this topic. Myotonometry is equally objective but cheaper and easier to carry out than the commonly performed imaging techniques and electrophysiological methods. Myotonometry provides information concerning the subject of the mechanical and visco-elastic properties of muscles. The MyotonPRO device (Myoton AS, Tallinn, Estonia) has emerged as a reliable device for testing the tone and properties, such as the elasticity or stiffness, of the masseter muscles [17,18].

In the literature available, the authors of the study reported herein did not find any studies evaluating the condition of the masseter muscles after orthodontic treatment by means of myotonometry. The aim of this study is to answer the question of whether the treatment of malocclusion with the use of fixed orthodontic appliances changes the mechanical and visco-elastic properties of the masseter muscles, in comparison with the same variables in healthy individuals who do not qualify for treatment.

## 2. Materials and Methods

### 2.1. Participants

The study included patients who met the following inclusion criteria: healthy adolescents and adults, normal craniofacial morphology [12], congenital as well as acquired malocclusion resulting in improper occlusion assessed using Angle’s classifications [19], bruxism, or the need to improve teeth aesthetics, and orthodontic treatment with the use of permanent appliances for a period of a min. of 6 and a max. of 18 months. The non-instrumental approach (self-report) was employed to assess bruxism in accordance with international consensus on the assessment of bruxism [20]. Grade 1: Possible episodes of sleep/awake bruxism is based on a positive self-report only.

Exclusion criteria included the occurrence of craniofacial anomalies as well as systemic diseases and incomplete dentition.

There were 74 study participants (40 females and 34 males) in the age range from 18 to 23 years, average age of 20.1 ± 1.7 years. The research material was divided into 2 groups. Group 1 consisted of 37 orthodontic patients including 4 patients with bruxism with fixed appliances installed for an average period of 12.27 ± 3.5 months. They were examined by an orthodontist and diagnosed with Angle Class II, i.e., distal occlusion, and Angle Class III, i.e., anterior occlusion. In total, 37 subjects were assigned to group 2 (control). They were also examined by the orthodontist and diagnosed with Angle Class I, i.e., normal occlusion.

### 2.2. Measurements

**Figure 1:**
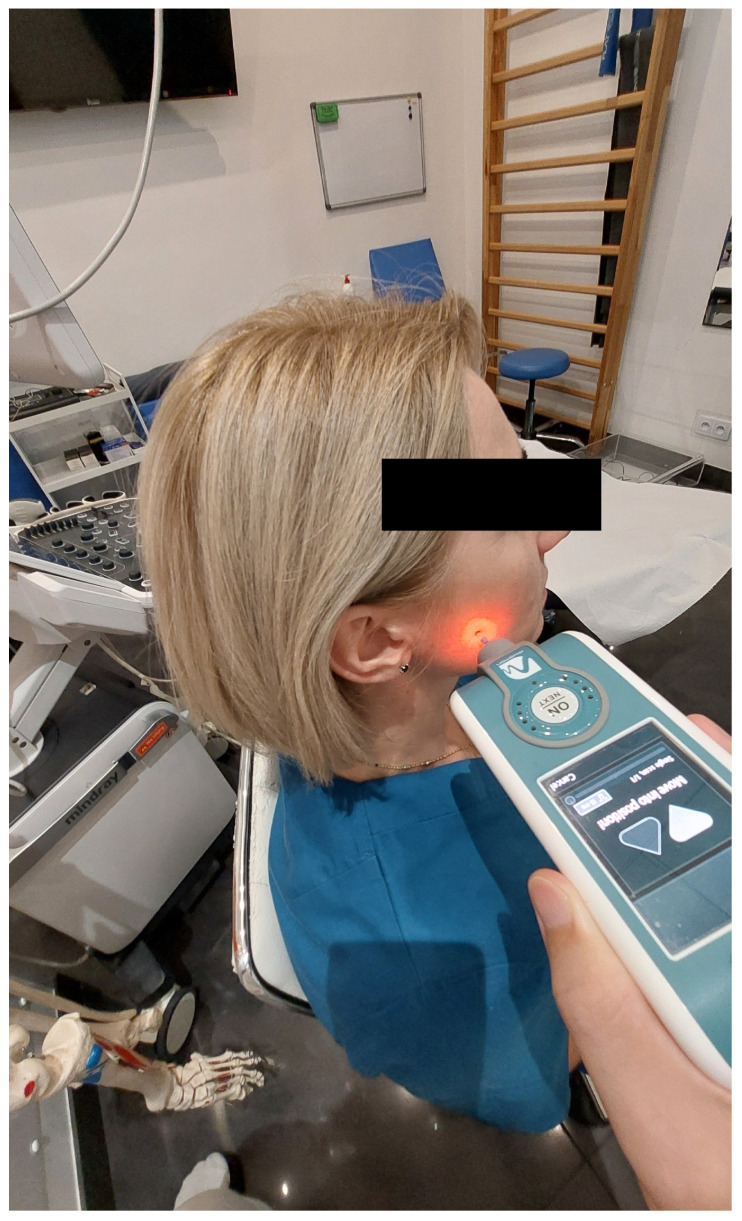
The MyotonPRO measurement technique.

The masseter muscles were assessed on both sides, with the patient sitting with their head in a neutral position. Myotonometer measurements were performed with the use of the MyotonPRO device (Myoton AS, Tallinn, Estonia) first under muscle relaxation conditions and then during maximum muscle contraction, during forceful biting [17]. The measurements were repeated twice, for the right and left sides. The results of the measurements were averaged separately for the right and left sides and for measurements taken in relaxation and maximum muscle contraction. During the examination, the coefficient of variation (CV) of each test result was taken note of, and in cases where the CV exceeded 3%, the test was repeated once again [21]. In order to determine the measurement point, a straight line was drawn from the participant’s eye corner (A) to the mandibular angulus (B) in the examination reported; also, the intersection point between the straight line (A-B) and the zygomatic bone was defined as C. The middle point of B-C, which was set as the measurement point for the masseter muscle (D), correlated with the midpoint of the masseter muscle belly [16]. The location of the measuring point thus determined was the anatomically largest cross-section of the muscle. The measurement point was marked with a marker before myotonometric measurements were performed. The MyotonPRO testing end was placed at the pre-designated point and marked with a marker, perpendicular to the skin surface (Figure 1). Once the device was stable in the measurement position, which was indicated by the control diode being “on”, automatic preloading of 0.18 N was used, and an automatic mechanical impulse was engaged for 15 ms, at the pre-determined force of 0.4 N (quick release). The oscillations of the examined tissue were recorded using an accelerometer. The following values were calculated: frequency (F) [Hz], identifying tone; stiffness (S) [N/m], indicating the ability of the tissue to oppose external forces that modify its shape; and decrement (D) [log], characterizing elasticity (the ability of a muscle to recover/regain its shape after deformation; the lower its value, the higher the elasticity and also the lower the damping of the tissue oscillation [22].

The criteria for the interpretation of the results were the following: the higher the values of (F) and (S), the greater the tone and stiffness of tissue; also, the lower the value of (D), the lower the dissipation of mechanical energy during oscillation and the higher the elasticity of tissue. MyotonPRO proves to be a reliable device and method for quantifying the stiffness of the masseter muscles and monitoring changes occurring in them under varying contraction conditions [16,17]. The intra-rater reliability turned out to be good (ICC = 0.78) and inter-operator reliability was reported to be excellent (ICC = 0.95) for making the assessment of masseter muscle stiffness with the use of MyotonPRO [17]. In all of the measurements taken by means of the MyotonPRO device as well as in qualification for the study, an experienced physiotherapist was involved, who had been trained in the field of myotonometry.

Myotonometric measurements of the masseter muscles were performed within 1–3 days after the end of treatment with fixed orthodontic appliances.

### 2.3. Statistical Analysis

The Statistica 13 package (Statsoft, Kraków, Poland) was applied to analyze the study data. The results were presented in the form of mean values, standard deviation, and confidence intervals. The distribution of the studied variables was examined by means of the Shapiro–Wilk test. Student’s *t*-test was used accordingly to test the statistical significance of differences between groups regarding the studied parameters. Qualitative variable evaluation was performed using the chi-square test. The level of statistical significance was assumed to be *p* < 0.05. G*power software (version 3.1.9.7; Heinrich-Heine-Universität Düsseldorf, Düsseldorf, Germany; (http://www.gpower.hhu.de, accessed on 20 May 2024) [23] was applied to determine the force using 2-sided testing, α err prob = 0.05, medium effect size d = 0.5, and a sample size of 37 patients in each group. After determining the mean values and standard deviation for the stiffness parameter in both groups, the force (1–err prob) was calculated as 0.72 and the effect sized = 0.60. Post hoc calculation was performed for the independent *t*-test.

## 3. Results

There were no statistically significant differences between groups 1 and 2 as regards age range (19.08 ± 0.71 vs. 21.18 ± 1.37; *p* = 0.372), BMI values (20.94 ± 4.5 vs. 22.13 ± 3.4; *p* = 0.103), and the number of men and women (14 men and 23 women in group 1 vs. 16 men and 21 women in group 2; *p* = 0.432). Statistical analysis of the results of myotonometric measurements showed that the values of the parameters of tension, stiffness, and elasticity of the masseter muscles did not statistically significantly differ between group 1 (treated using fixed orthodontic appliances) and group 2 (control—not subjected to orthodontic treatment). This concerned both the examination of the muscles in relaxation (Table 1) and the masseter muscles at the maximum contraction, during forceful biting (Table 2). In group 1, no statistically significant differences between the right and left masseter muscles were found, when tested in relaxation conditions, as regards tension (*p* = 0.739), stiffness (*p* = 0.858), and elasticity (*p* = 0.407), nor were they found at maximum contraction conditions, as regards tension (*p* = 0.450), stiffness (*p* = 0.455), and elasticity (*p* = 0.383).

**Figure 2:**
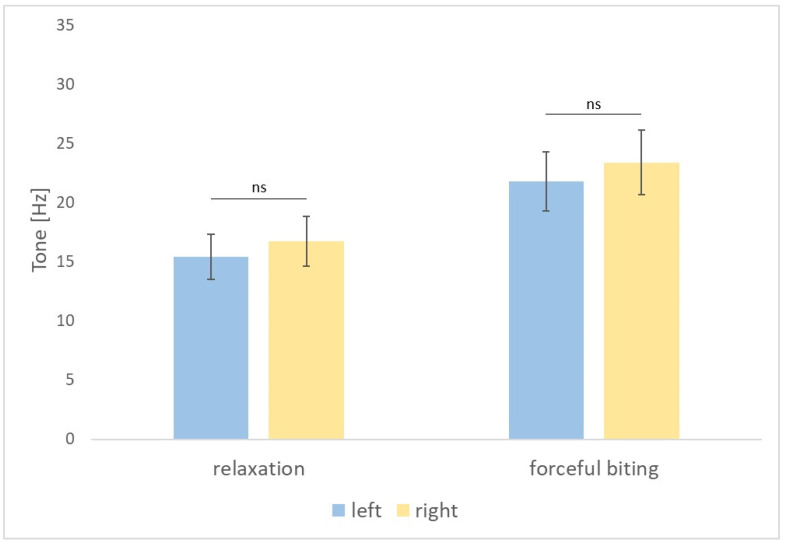
Tone of the masseter muscles on the left and right side measured in relaxation and during forceful biting. *p*-value > 0.05 is indicated by “ns” for not significant.

**Figure 3:**
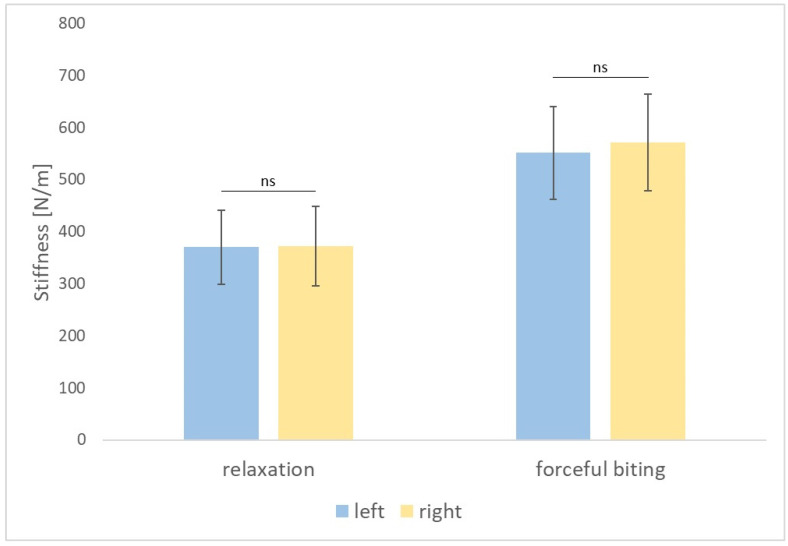
Stiffness of the masseter muscles on the left and right side measured in relaxation and during forceful biting. *p*-value > 0.05 is indicated by “ns” for not significant.

**Figure 4:**
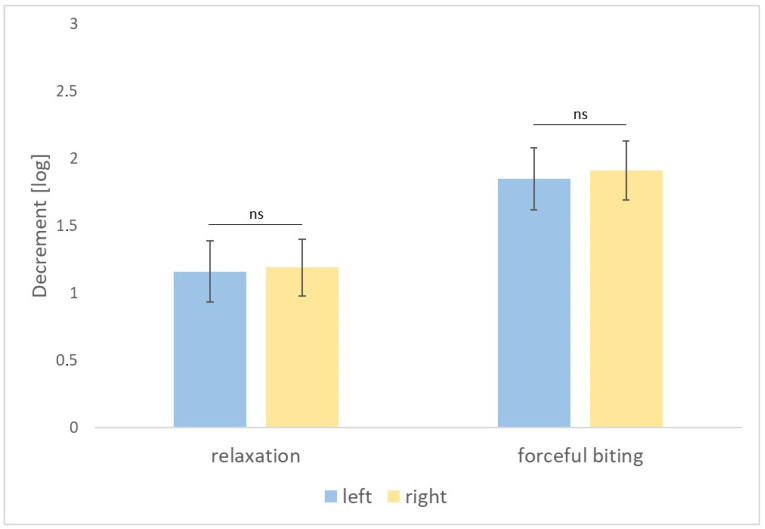
Decrement of the masseter muscles on the left and right side measured in relaxation and during forceful biting. *p*-value > 0.05 is indicated by “ns” for not significant.

In group 1, no statistically significant differences were found between the right and left masseter muscles tested in relaxation as regards tension (*p* = 0.739), stiffness (*p* = 0.858), and elasticity (*p* = 0.407) and at maximum contraction, during forceful biting as regards tension (*p* = 0.450), stiffness (*p* = 0.455), and elasticity (*p* = 0.383) (Figure 2, Figure 3 and Figure 4).

## 4. Discussion

Muscles play an important role in maintaining the proper functioning of the chewing apparatus, and bite strength and the efficiency of the masseter muscles are closely dependent on each other [24]. In the study presented herein, the MyotonPRO device was used to examine masseter muscles after the completion of orthodontic treatment. The most important findings concerning the biomechanical attributes of the masseter muscles indicate the proper condition of the main masticatory muscle after the application of a fixed orthodontic appliance. The assessed parameters of tension, stiffness, and elasticity did not differ with statistical significance between the group of patients treated by means of a fixed appliance and the control group not undergoing orthodontic treatment. Physiologically, this may indicate that there were no significant changes in neuromuscular excitability, as an indicator of the immediate adaptive response of muscle fibers, as well as structural changes related to connective tissue (fascia) remodeling.

Maximum bite force (MBF) is treated as the major indicator of chewing function and is supposed to be correlated with the performance of the masseter muscles. On the other hand, objective measurement of masseter muscle stiffness by means of MyotonPRO, which has high test–retest reliability, remains significantly correlated with muscle strength [17].

According to our knowledge, no previous study has examined the relationship between the use of fixed orthodontic appliances and the condition of the masseter muscles described objectively by means of quantitative data. On the other hand, there are numerous studies assessing the strength of bite after orthodontic treatment, which directly depends on the functional condition of the masseter muscles. In study [16], it was demonstrated that reduced maximum bite force (MBF) is caused not only by typical dental disorders, such as reduced occlusive contact area, loss of teeth, or type of artificial denture, but also by disorders of masseter muscle function, resulting in the weakening of muscle strength assessed by examinations directly, as in the case of bite force measurement, or indirectly by means of measuring the cross-sectional area (CSA) and myotonometry. Naturally, muscle weakness may follow from the above-mentioned dental disorders, but it may also be the result of primary muscle dysfunctions. The authors recommend that masseter muscle strength and typical dental dysfunction be assessed separately when evaluating MBF.

Muscle stiffness, as one of the parameters assessed in the myotonometric examination performed at the maximum of any muscle contraction or relaxation is, according to literature data, a recognized indirect indicator of muscle strength [16,17]. Muscle stiffness increases in line with the increase in the strength of contraction. Therefore, it is assumed that the value of muscle stiffness reflects the strength generated by the masseter muscle when biting strongly. When stiffness increases, tension also increases, while the elasticity of the muscle decreases. When a single muscle fiber shrinks, its thickness and cross-sectional area increase. The increase in thickness and cross-sectional area of the muscle is in proportion to the increase in tension. The difference in the thickness of the masseter muscle is considered to be a proper indication of the tension and activity level of the masseter muscle, evaluated by means of electromyography (EMG). These findings show that stiffness and tension measurements have the potential for the assessment of the force generated and the activation of the masseter muscles during strong biting [25,26].

In the study conducted by Yu et al. [17], in healthy individuals, the average masseter muscle stiffness assessed by means of MyotonPRO amounted to 369.5 ± 94.9 N/m at relaxation, compared with 378.68 ± 71.55 N/m in healthy individuals in our study, and 618.3 ± 150.7 N/m at maximum contraction, as compared to 618.2 ± 101.49 N/m in our study. The increase in stiffness reported in that study, at maximum muscle contraction, by 64.7% in comparison with relaxation, was comparable to the increase of 63.25% observed in our study. Masseter muscles have an important role to play in balancing chewing function, while the increase in muscle stiffness turns out to improve the stability of chewing movements when modulating bite forces [27].

Another study showed that the thickness of the masseter muscles correlates positively with bite force [25]. Moreover, in the case of normal anatomy conditioning optimal biomechanics, the right and left masseter muscles should be equally involved in maintaining normal chewing function. This is confirmed by the results of both our study and other studies employing myotonometry [17], ultrasonography [28], shear wave elastography (SWE) [29], and surface electromyography (sEMG) [30].

MyotonPRO is a cheap, portable, and easy-to-use device for testing muscle biomechanical parameters that can exceed the value of EMG examinations, with high repeatability of the results of the former, with limitations of EMG resulting from the fact that the electrical signal is susceptible to numerous disturbances. In addition, myotonometry is an examination method that is many times cheaper than shear wave elastography (SWE) which is also used for the examination of muscle stiffness.

## 5. Limitations of the Study

Although our study resulted in new findings, it also has some limitations. This study deals with the question concerning the biomechanical parameters of masseter muscle change after the use of a fixed orthodontic appliance, as compared to healthy individuals who do not qualify for orthodontic treatment. On the other hand, our study does not answer the question concerning the impact of the use of fixed orthodontic appliances on the biomechanical properties of the masseter muscles because the authors did not compare the biomechanical parameters of muscles before, during, and after treatment. It is necessary to plan such a study in the future. Also, the examination was performed at one standardized site of the masseter muscle but may not be representative of the overall stiffness of the entire muscle. The maximum contraction of the masseter muscles was defined subjectively. Therefore, future studies appear to be necessary to quantify maximum masseter muscle contraction. In addition, it should be taken into account that maximum bite force is related to overall physical strength, which may affect the results obtained.

## 6. Conclusions

This study reveals that there are no significant differences in the biomechanical and visco-elastic properties of masseter muscles assessed by means of myotonometry, between the patients treated with fixed appliances due to malocclusions and those not requiring orthodontic treatment. To confirm our initial results, the study should be repeated on a larger sample.

## Figures and Tables

**Table 1:** The scores in the myotonometric outcomes of the left and right masseter muscles in relaxation.

	Group 1				Group 2				
	Mean	SD	−95% CI	95% CI	Mean	SD	−95% CI	95% CI	*p*-Level
F (L)	15.41	1.90	14.79	16.02	15.93	1.94	15.31	16.55	0.612
S (L)	370.03	70.98	347.16	392.91	381.40	74.28	357.46	405.33	0.248
D (L)	1.16	0.23	1.08	1.24	1.19	0.25	1.11	1.27	0.583
F (R)	16.74	2.11	16.06	17.42	16.68	1.96	16.05	17.31	0.891
S (R)	371.82	76.09	347.30	396.33	375.97	68.83	353.79	398.15	0.609
D (R)	1.19	0.21	1.13	1.26	1.22	0.21	1.15	1.29	0.604

Abbreviations used: F—Frequency [Hz], S—Stiffness [N/m], D—Decrement [log], (L)—left side, and (R)—right side.

**Table 2:** The scores in the myotonometric outcomes of the left and right masseter muscles at maximum contraction, during forceful biting.

	Group 1				Group 2				
	Mean	SD	−95% CI	95% CI	Mean	SD	−95% CI	95% CI	*p*-Level
F (L)	21.78	2.48	20.98	22.58	23.87	2.09	23.20	24.55	0.305
S (L)	551.52	89.27	522.76	580.28	606.23	99.27	574.25	638.22	0.073
D (L)	1.85	0.23	1.78	1.92	1.92	0.30	1.82	2.02	0.449
F (R)	23.39	2.71	22.52	24.27	26.11	2.26	25.38	26.84	0.187
S (R)	571.02	92.99	541.05	600.98	630.17	103.72	596.75	663.59	0.061
D (R)	1.91	0.22	1.84	1.98	2.07	0.20	2.01	2.14	0.296

Abbreviations: F—Frequency [Hz], S—Stiffness [N/m], D—Decrement [log], (L)—left side, and (R)—right side.

## Data Availability

The datasets used and/or analyzed during the current study are available from the corresponding author upon reasonable request.
